# Supplementary material for: Co-existing feedback loops generate tissue-specific circadian rhythms
Source: Life Sci Alliance. 2018 Jun 14;1(3):e201800078. doi: 10.26508/lsa.201800078 (PMC6238625; doi:10.26508/lsa.201800078)
Supplement: Supplementary file 1 [file LSA-2018-00078_Supplementary_Information_1.pdf]

S1 Model equations

Underlying mathematical model

For fitting experimental data we start with a network model that was introduced by Korenčič et al. in 2014 [1]. It uses only 5 variables representing groups of genes with distinct expression peaks. Genes with the same peak time and binding mechanism of their protein products are modeled by the same variable. For example, all early E-box inhibitors, such as *Per1*, *Per2*, *Per3* and *Cry2* are represented by a single variable called *Per2*. The late E-box inhibitor *Cry1*, however, is modeled as a second variable.

Further, the model consists of Delay-Differential Equations (DDE). Regulations affects other genes only after certain time delays, which are parameters in the model. A time delay could correspond to time consuming processes such as nuclear translocation or complex formation and is important for oscillations with a circadian period. Models using Ordinary Differential Equations (ODE) require additional intermediate steps to obtain the right temporal scales. Thus, use of DDE also allows to represent the core clock network with a small number of variables and parameters.

Taken together, the model constitutes a condensed representation of the core clock network. It is not targeted at capturing all mechanistic details, but rather presents a regression that exploits timing of gene expression to understand self-sustained circadian oscillations.

Structure of Equations

The full set of equations is shown in Example **S1-1**. Note the modular structure of activatory and inhibitory terms, marked with blue and red labels respectively. Note, that activator terms contain two free parameters (maximum fold induction and activation threshold), whereas inhibitory terms have just a single free parameter—the inhibition threshold. The interactions of the model were assembled in a comprehensive survey of ChIP-Seq experiments and motif mapping. The number of binding sites of a gene is reflected in the power of the corresponding term.

References

[1] Korenčič A, Košir R, Bordyugov G, Lehmann R, Rozman D, Herzel H. Timing of circadian genes in mammalian tissues. Sci Rep. 2014;4:5782.

Example S1-1 Full set of Delay-Differential Equations (DDEs) of the circadian core clock model.

$$\frac{d\,[Bmal1]}{dt} = \underbrace{\left(\frac{1}{\frac{[\mathrm{RevErb}_\alpha](\mathrm{t{-}del2})}{\mathrm{ar1}}+1}\right)^2}_{\text{Rev-erb-}\alpha\text{ (-)}} -\mathrm{d1}\,[Bmal1]\,(t)$$

$$\frac{d\,[RevErb_\alpha]}{dt} = \underbrace{\left(\frac{\frac{\mathrm{b\_RevErb}\,[\mathrm{Bmal1}](\mathrm{t{-}del1})}{\mathrm{ba2}}+1}{\frac{[\mathrm{Bmal1}](\mathrm{t{-}del1})}{\mathrm{ba2}}+1}\right)^3}_{\text{Bmal1 (+)}} \underbrace{\left(\frac{1}{\frac{[\mathrm{Per2}](\mathrm{t{-}del3})}{\mathrm{cr2}}+1}\right)^3}_{\text{Per2 (-)}}$$
$$\underbrace{\left(\frac{\frac{\mathrm{f\_RevErb}\,[\mathrm{Dbp}](\mathrm{t{-}del5})}{\mathrm{fa2}}+1}{\frac{[\mathrm{Dbp}](\mathrm{t{-}del5})}{\mathrm{fa2}}+1}\right)}_{\text{Dbp (+)}} \underbrace{\left(\frac{1}{\frac{[\mathrm{Cry1}](\mathrm{t{-}del4})}{\mathrm{gr2}}+1}\right)^3}_{\text{Cry1 (-)}} -\mathrm{d2}\,[RevErb_\alpha]\,(t)$$

$$\frac{d\,[Per2]}{dt} = \underbrace{\left(\frac{\frac{\mathrm{b\_Per2}\,[\mathrm{Bmal1}](\mathrm{t{-}del1})}{\mathrm{ba3}}+1}{\frac{[\mathrm{Bmal1}](\mathrm{t{-}del1})}{\mathrm{ba3}}+1}\right)^2}_{\text{Bmal1 (+)}} \underbrace{\left(\frac{1}{\frac{[\mathrm{Per2}](\mathrm{t{-}del3})}{\mathrm{cr3}}+1}\right)^2}_{\text{Per2 (-)}} \underbrace{\left(\frac{\frac{\mathrm{f\_Per2}\,[\mathrm{Dbp}](\mathrm{t{-}del5})}{\mathrm{fa3}}+1}{\frac{[\mathrm{Dbp}](\mathrm{t{-}del5})}{\mathrm{fa3}}+1}\right)}_{\text{Dbp (+)}} \underbrace{\left(\frac{1}{\frac{[\mathrm{Cry1}](\mathrm{t{-}del4})}{\mathrm{gr3}}+1}\right)^2}_{\text{Cry1 (-)}} -\mathrm{d3}\,[Per2]\,(t)$$

$$\frac{d\,[Cry1]}{dt} = \underbrace{\left(\frac{1}{\frac{[\mathrm{RevErb}_\alpha](\mathrm{t{-}del2})}{\mathrm{ar4}}+1}\right)^2}_{\text{Rev-erb-}\alpha\text{ (-)}} \underbrace{\left(\frac{\frac{\mathrm{b\_Cry1}\,[\mathrm{Bmal1}](\mathrm{t{-}del1})}{\mathrm{ba4}}+1}{\frac{[\mathrm{Bmal1}](\mathrm{t{-}del1})}{\mathrm{ba4}}+1}\right)^2}_{\text{Bmal1 (+)}}$$
$$\underbrace{\left(\frac{1}{\frac{[\mathrm{Per2}](\mathrm{t{-}del3})}{\mathrm{cr4}}+1}\right)^2}_{\text{Per2 (-)}} \underbrace{\left(\frac{\frac{\mathrm{f\_Cry1}\,[\mathrm{Dbp}](\mathrm{t{-}del5})}{\mathrm{fa4}}+1}{\frac{[\mathrm{Dbp}](\mathrm{t{-}del5})}{\mathrm{fa4}}+1}\right)}_{\text{Dbp (+)}} \underbrace{\left(\frac{1}{\frac{[\mathrm{Cry1}](\mathrm{t{-}del4})}{\mathrm{gr4}}+1}\right)^2}_{\text{Cry1 (-)}} -\mathrm{d4}\,[Cry1]\,(t)$$

$$\frac{d\,[Dbp]}{dt} = \underbrace{\left(\frac{\frac{\mathrm{b\_Dbp}\,[\mathrm{Bmal1}](\mathrm{t{-}del1})}{\mathrm{ba5}}+1}{\frac{[\mathrm{Bmal1}](\mathrm{t{-}del1})}{\mathrm{ba5}}+1}\right)^3}_{\text{Bmal1 (+)}} \underbrace{\left(\frac{1}{\frac{[\mathrm{Per2}](\mathrm{t{-}del3})}{\mathrm{cr5}}+1}\right)^3}_{\text{Per2 (-)}} \underbrace{\left(\frac{1}{\frac{[\mathrm{Cry1}](\mathrm{t{-}del4})}{\mathrm{gr5}}+1}\right)^3}_{\text{Cry1 (-)}} -\mathrm{d5}\,[Dbp]\,(t)$$
