# Supplementary material for: Co-existing feedback loops generate tissue-specific circadian rhythms
Source: Life Sci Alliance. 2018 Jun 14;1(3):e201800078. doi: 10.26508/lsa.201800078 (PMC6238625; doi:10.26508/lsa.201800078)
Supplement: Supplementary file 2 [file LSA-2018-00078_Supplementary_Information_3.pdf]

S3 Scoring parameter sets for global optimization

Extracting features from experimental data

We fitted harmonic models to experimental data [1] that is publicly available for different tissues with a circadian resolution of 2 h over two days. Using such models, measurements are approximated to yield more reliable estimates of amplitudes and phases.

The fitted models have the form given in Equation **S3-1**.

Thus, the functions contain harmonics and have curve shapes with wide troughs and narrow peaks. Such a shape is well suited for the measured time series.

The fit parameters a, b and c are obtained by nonlinear regression. Example fits of harmonic models to liver data are shown in Figure **S3-1**.

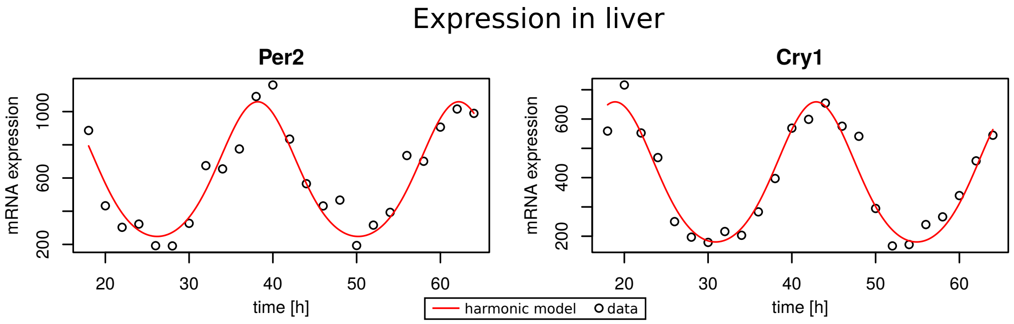

Figure S3-1: Fitted harmonic models and data points for *Per2* and *Cry1* in mouse liver.

Equation S3-1 Fitted harmonic model

$$f(t) = \left[a \cdot \sin\left(\frac{2\pi t}{24}\right) + b \cdot \cos\left(\frac{2\pi t}{24}\right) + c\right]^4$$

Equation S3-2 Scoring function

$$score = \frac{(period_{sim} - period_{exp})^2}{{tol_{period}}^2} + \sum \frac{(phase_{sim} - phase_{exp})^2}{{tol_{phase}}^2} + \sum \frac{(foldch_{sim} - foldch_{exp})^2}{{tol_{foldch}}^2}$$

The scoring function

The complete scoring function incorporating period, phases and fold changes is given in Equation **S3-2**. Differences between simulated ($\cdot_{sim}$) and experimentally measured values ($\cdot_{exp}$) are weighted by tolerances ($tol_{\cdot}$).

As $phase_{sim}$ and $phase_{exp}$ relative phase differences to *Bmal1* are used. Thus, there are four phase differences.

The fold changes are calculated as $\log_2 \frac{max}{min}$. For experimental values we use maxima and minima (peaks and troughs) derived from harmonic fits to the data. In this way, measurement errors of individual points at peaks or troughs are reduced since all points contribute to the fits.

There are 10 terms in total (1 period + 4 phases + 5 amplitudes). If differences between data and fit are equal to the tolerances we get a score of 10. Thus, we consider a score of 10 as a reasonable cutoff. Figure **S3-2** shows an example fit with a score close to 10.

Tolerances

We use a tolerance of $tol_{period} = 0.1$ h for the period, reflecting typical experimental deviations in mice WT data. For relative phases we compare measurements from different experiments [1–3] and derive a tolerance of $tol_{phase} = 1$ h. Figure **S3-3** shows that relative phases measured in the three experiments are indeed comparable.

Fold changes are less consistent between the different experiments. To define a reasonable tolerance we therefore split the Zhang et al. data set into day 1 and day 2 and compare the deviation between these days. Since fold changes vary strongly between genes, we define five tolerances (one for each gene) based on the median differences between the days: $tol_{foldch(Bmal1)} = 0.1$, $tol_{foldch(Reverba)} = 0.4$, $tol_{foldch(Per2)} = 0.2$, $tol_{foldch(Cry1)} = 0.1$, $tol_{foldch(Dbp)} = 0.12$.

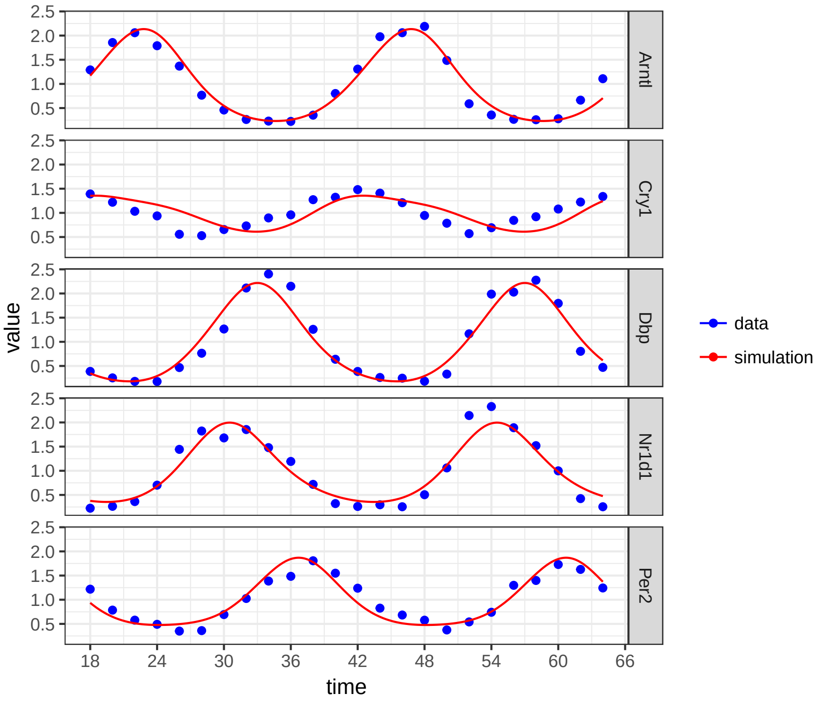

Figure S3-2: Example fit to kidney-data with a score of 9.525. Data points and simulation are shown in blue and red respectively.

Cuts through the fitness landscape

Application of the scoring function to all parameter combinations yields a 35 dimensional landscape with troughs and peaks. Using global optimization with VFO (see Supplement S4) and Particle Swarm Optimization we search troughs with minimal score.

In Figure S3-4 we present a representative selection of cuts through the fitness landscape along one parameter axis. For each depicted parameter, cuts are shown for models fitted to SCN and liver. The parameter values are taken from successful optimization runs and the found minimum is marked in red.

This depiction presents useful information to judge the quality of fits and the ability to identify unique parameter values. Most cuts have a near-parabolic shape and clear trough as in the first 2 columns of Figure S3-4. Only some cuts have minima located in less steep troughs, as for example in row 1, column 3. In fits with less good scores, as for example in row 1, sometimes also a boundary is reached after which rhythms vanish (dotted lines). In just a few cases there is no clear optimum (e.g. last plot in row 1).

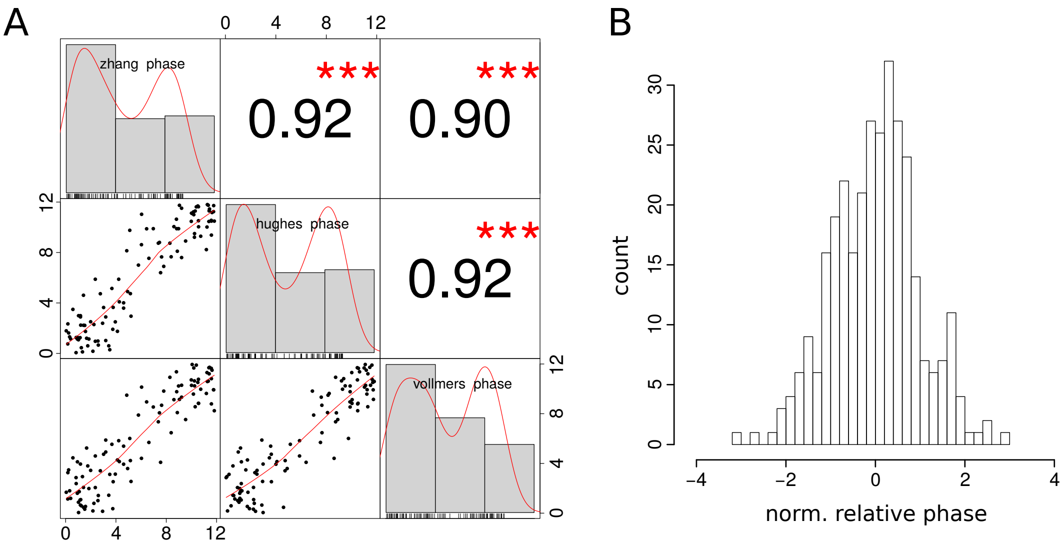

Figure S3-3: (A) Correlations of relative phase relationships across three experiments showing Pearson correlation coefficients. (B) Histogram of phase differences to the gene-specific mean value. The standard deviation is about 1 h.

Interestingly, *Cry1* ⊣ *Per2*, a regulation that is part of the repressilator, has a clear trough for the fit to liver data which contains a repressilator in its rhythm generating set of loops. However, it shows no discernible optimum for the SCN fit that does not involve this regulation as an essential part of its oscillation generating mechanism.

References

[1] Zhang R, Lahens NF, Ballance HI, Hughes ME, Hogenesch JB. A circadian gene expression atlas in mammals: implications for biology and medicine. Proc Natl Acad Sci USA. 2014;111(45):16219–16224.

[2] Hughes ME, DiTacchio L, Hayes KR, Vollmers C, Pulivarthy S, Baggs JE, et al. Harmonics of circadian gene transcription in mammals. PLoS Genet. 2009;5(4):e1000442.

[3] Vollmers C, Gill S, DiTacchio L, Pulivarthy SR, Le HD, Panda S. Time of feeding and the intrinsic circadian clock drive rhythms in hepatic gene expression. Proc Natl Acad Sci USA. 2009;106(50):21453–21458.

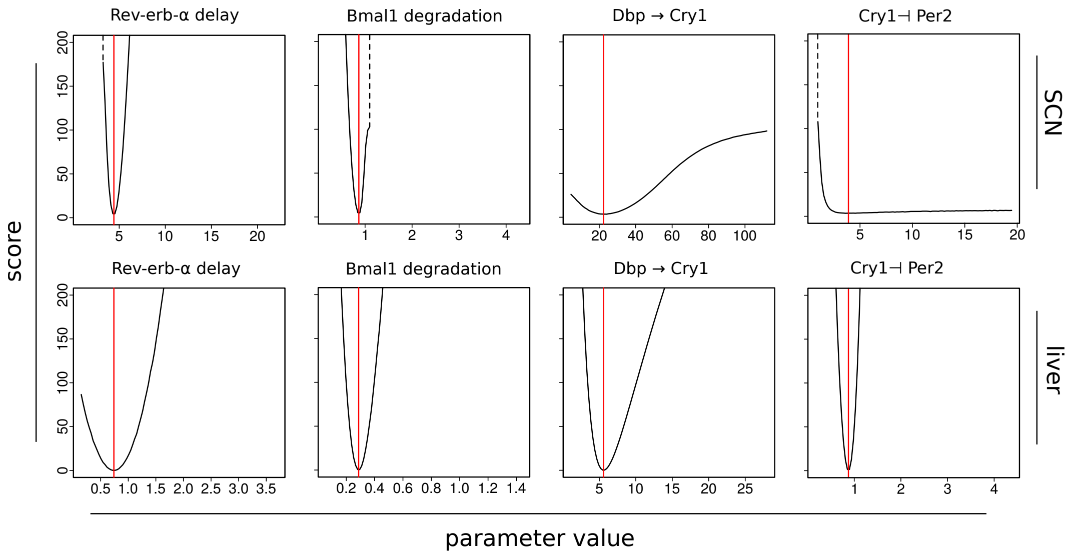

Figure S3-4: Cuts through the 35 dimensional fitness landscape created by applying the scoring function (Eq. **S3-2**) to combinations of 34 parameters. In each plot one parameter of an optimal model fit is varied from 0.2 to 5 times the optimal value (red line). Transitions to regions with no oscillation are marked by dotted lines.
