# Supplementary material for: Co-existing feedback loops generate tissue-specific circadian rhythms
Source: Life Sci Alliance. 2018 Jun 14;1(3):e201800078. doi: 10.26508/lsa.201800078 (PMC6238625; doi:10.26508/lsa.201800078)
Supplement: Supplementary file 3 [file LSA-2018-00078_Supplementary_Information_2.pdf]

S2 Parameter sets

Here we list sample parameter sets for each tissue with their score and minimal oscillators. The score was given according to the scoring function in Supplement S3. "Minimal oscillator" refers to the finding, that the listed loops can synergistically generate oscillations, if they are not clamped. Minimal means that at least these loops need to be active. The models shown here are taken from the top scoring fits of each tissue.

adrenal gland

score: 0.091
oscillator: *Bmal1*/*Rev-erb-α* + Repressilator

del1	del2	del3	del4	del5	d1	d2	d3	d4	d5
5.06	0.49	2.88	5.55	5.28	0.30	0.48	0.26	0.56	0.33

ar1	ar4	cr2	cr3	cr4	cr5	gr2	gr3	gr4	gr5	b_RevErb	ba2	b_Per2	ba3
1.29	9.02	1.69	7.16	2.99	1.87	4.50	0.38	0.98	0.40	3.65	0.37	1.15	2.36

b_Cry1	ba4	b_Dbp	ba5	fa2	f_RevErbA	fa3	d_Per2	fa4	d_Cry1
1.17	11.34	6.56	0.19	42.91	4.65	6.06	5.08	1.00	4.29

kidney

score: 0.090

oscillator 1: Bmal1/Reverba + Per2 + Per2/Dbp + Repressilator

oscillator 2: Bmal1/Reverba + Per2/Dbp + Cry1 + Repressilator

oscillator 3: Bmal1/Reverba + Per2/Dbp + Cry1/Dbp + Repressilator

del1	del2	del3	del4	del5	d1	d2	d3	d4	d5
5.65	0.88	5.34	4.81	4.50	0.50	0.73	0.41	0.27	0.43

ar1	ar4	cr2	cr3	cr4	cr5	gr2	gr3	gr4	gr5	b_RevErb	ba2	b_Per2	ba3
1.51	2.99	1.92	4.82	1.95	0.71	4.04	0.23	0.19	0.42	2.69	0.34	1.50	9.62

b_Cry1	ba4	b_Dbp	ba5	fa2	f_RevErbA	fa3	d_Per2	fa4	d_Cry1
1.23	0.82	3.50	0.04	12.02	2.09	4.22	5.87	1.55	4.25

liver

score: 0.014

oscillator 1: Bmal1/Reverba + Per2/Dbp + Repressilator

oscillator 2: Bmal1/Reverba + Cry1 + Repressilator

del1	del2	del3	del4	del5	d1	d2	d3	d4	d5
5.83	0.74	2.74	3.35	5.29	0.29	0.77	0.42	0.23	0.42

ar1	ar4	cr2	cr3	cr4	cr5	gr2	gr3	gr4	gr5	b_RevErb	ba2	b_Per2	ba3
1.83	4.40	0.84	14.82	12.16	3.16	8.03	0.87	1.83	0.67	4.06	0.12	1.81	3.45

b_Cry1	ba4	b_Dbp	ba5	fa2	f_RevErbA	fa3	d_Per2	fa4	d_Cry1
1.66	0.52	4.40	0.90	12.68	2.61	1.74	4.03	4.15	5.59

SCN

score: 3.49

oscillator: Per2/Dbp + Cry1 + Cry1/Dbp

del1	del2	del3	del4	del5	d1	d2	d3	d4	d5
5.44	3.87	4.25	5.83	3.72	0.39	0.49	0.45	0.10	0.32

ar1	ar4	cr2	cr3	cr4	cr5	gr2	gr3	gr4	gr5	b_RevErb	ba2	b_Per2	ba3
5.86	14.88	0.38	5.63	5.80	6.39	10.22	0.06	0.07	0.23	1.45	0.22	10.96	8.04

b_Cry1	ba4	b_Dbp	ba5	fa2	f_RevErbA	fa3	d_Per2	fa4	d_Cry1
7.43	7.23	20.87	7.75	6.83	1.96	11.00	29.07	5.36	20.75

heart

score: 0.19

oscillator: Bmal1/Reverba

del1	del2	del3	del4	del5	d1	d2	d3	d4	d5
5.62	1.47	3.87	2.37	4.46	0.36	0.46	0.95	0.22	0.92

ar1	ar4	cr2	cr3	cr4	cr5	gr2	gr3	gr4	gr5	b_RevErb	ba2	b_Per2	ba3
3.52	20.43	9.97	5.68	11.03	2.56	4.09	0.11	0.18	0.05	5.71	1.00	4.39	7.50

b_Cry1	ba4	b_Dbp	ba5	fa2	f_RevErbA	fa3	d_Per2	fa4	d_Cry1
3.69	12.35	10.62	0.07	21.22	2.85	15.87	16.60	4.75	5.31

skeletal muscle

score: 1.1

oscillator 1: Bmal1/Reverba + Per2

oscillator 2: Bmal1/Reverba + Per2 + Per2/Dbp

oscillator 3: Bmal1/Reverba + Per2 + Cry1

oscillator 4: Bmal1/Reverba + Per2/Dbp + Cry1

del1	del2	del3	del4	del5	d1	d2	d3	d4	d5
4.08	4.58	5.98	4.76	3.37	0.72	0.94	0.35	0.37	0.16

ar1	ar4	cr2	cr3	cr4	cr5	gr2	gr3	gr4	gr5	b_RevErb	ba2	b_Per2	ba3
5.54	18.49	12.63	1.15	6.96	0.19	5.32	0.94	2.89	11.30	13.03	4.74	1.00	14.65

b_Cry1	ba4	b_Dbp	ba5	fa2	f_RevErbA	fa3	d_Per2	fa4	d_Cry1
3.60	9.70	11.95	0.18	6.88	4.22	30.74	21.44	32.56	13.75

brown adipose

score: 0.276

oscillator: Bmal1/Reverba

del1	del2	del3	del4	del5	d1	d2	d3	d4	d5
5.60	0.52	2.92	4.27	4.77	0.23	0.84	0.71	0.28	0.32

ar1	ar4	cr2	cr3	cr4	cr5	gr2	gr3	gr4	gr5	b_RevErb	ba2	b_Per2	ba3
2.48	87.53	3.71	2.79	0.19	1.67	9.08	0.15	2.96	0.44	10.62	0.88	1.04	6.23

b_Cry1	ba4	b_Dbp	ba5	fa2	f_RevErbA	fa3	d_Per2	fa4	d_Cry1
1.38	7.26	22.54	0.14	22.34	3.75	10.43	4.13	10.39	4.25

white adipose

score: 0.171

oscillator1: Bmal1/Reverba + Per2/Dbp + Repressilator

oscillator2: Bmal1/Reverba + Cry1 + Repressilator

del1	del2	del3	del4	del5	d1	d2	d3	d4	d5
5.88	2.05	5.55	3.36	4.67	0.85	0.82	0.39	0.41	0.29

ar1	ar4	cr2	cr3	cr4	cr5	gr2	gr3	gr4	gr5	b_RevErb	ba2	b_Per2	ba3
2.96	7.38	1.70	4.73	14.88	0.25	3.07	0.24	0.31	1.89	3.46	0.45	2.10	6.46

b_Cry1	ba4	b_Dbp	ba5	fa2	f_RevErbA	fa3	d_Per2	fa4	d_Cry1
1.04	5.69	8.07	0.06	3.43	1.51	6.83	6.44	3.31	2.79

lung

score: 0.34

oscillator: Bmal1/Reverba

del1	del2	del3	del4	del5	d1	d2	d3	d4	d5
5.50	0.59	3.01	2.88	5.32	0.18	0.78	0.45	0.23	0.34

ar1	ar4	cr2	cr3	cr4	cr5	gr2	gr3	gr4	gr5	b_RevErb	ba2	b_Per2	ba3
1.11	29.82	1.60	10.51	9.90	9.73	7.46	0.24	0.22	0.40	7.21	0.54	1.84	7.92

b_Cry1	ba4	b_Dbp	ba5	fa2	f_RevErbA	fa3	d_Per2	fa4	d_Cry1
2.02	9.49	12.53	0.59	11.62	3.01	4.32	7.17	1.23	6.63

cerebellum

score: 0.545

oscillator: Bmal1/Reverba + Cry1

del1	del2	del3	del4	del5	d1	d2	d3	d4	d5
5.34	0.78	2.28	4.93	4.65	0.21	0.51	0.45	0.30	0.49

ar1	ar4	cr2	cr3	cr4	cr5	gr2	gr3	gr4	gr5	b_RevErb	ba2	b_Per2	ba3
7.18	28.26	23.94	2.43	4.97	16.79	11.16	0.09	0.53	0.69	15.27	8.15	1.02	11.15

b_Cry1	ba4	b_Dbp	ba5	fa2	f_RevErbA	fa3	d_Per2	fa4	d_Cry1
1.72	11.22	12.75	8.61	16.32	2.68	4.91	24.98	0.83	9.36
