# Supplementary material for: Co-existing feedback loops generate tissue-specific circadian rhythms
Source: Life Sci Alliance. 2018 Jun 14;1(3):e201800078. doi: 10.26508/lsa.201800078 (PMC6238625; doi:10.26508/lsa.201800078)
Supplement: Supplementary file 4 [file LSA-2018-00078_Supplementary_Information_5.pdf]

S5 Identification of essential feedback loops

Clamping regulations

Given a fitted parameter set, we analyze which loops in the network are essential for the generation of rhythms. To this end we employ our clamping strategy that was published earlier [1]: By setting combinations of regulatory links in the network graph to a constant value we systematically test which links are necessary for oscillations.

Let us assume that a regulatory link is part of an essential loop. Then rhythms vanish if we set this regulation to a constant. We choose the mean values of oscillations in the default state as a constant to preserve the basal effect exerted by the clamped regulation.

Any combination of regulations can be clamped by setting the respective parts in the differential equations constant.

Combinatorial exploration of the loop structure

To avoid excessive computation times, we do not test all combinations of regulations (which are $2^{17} = 131072$). Instead we resort to a targeted clamping strategy.

It is known that a negative feedback loop is necessary to generate oscillations [2]. Therefore, we list and test negative feedback loops in the network specifically.

We start by testing each single loop individually: A negative feedback loop is termed essential, if separate clamping of all regulations involved in the loop (i.e. clamping one regulation at a time) leads to disruption of rhythmicity. (Note, that in theory there might be a case where all edges of a negative feedback loop are shared by other loops, making it impossible to attribute the effect of clamping in that way, but this is not the case in our network.)

After testing single loops we proceed by testing combinations of loops. This is of interest, since loops could mutually compensate for each other. Then clamping just a single loop does not stop rhythms, because the other loop still

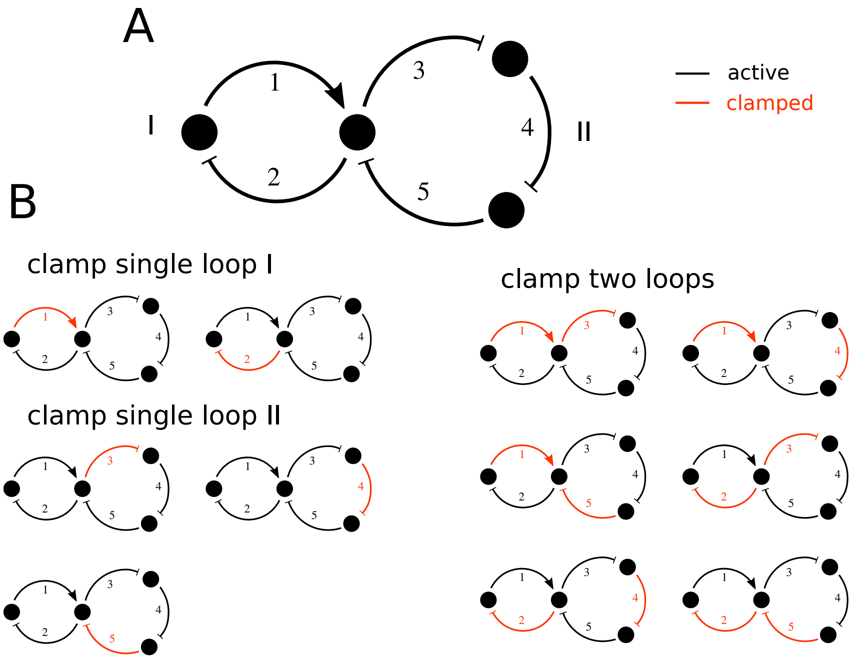

Figure S5-1: Example for targeted clamping of loops. (A) A toy network with two negative feedback loops ({1,2} and {3,4,5}). Assume that the model represented by this network is rhythmic, then both loops are candidates for the core mechanism generating these rhythms. The mechanism could involve loop I, loop II or both. (B) On the left: Clamping of single loops. To test whether e.g. loop II is essential, the model is simulated three times, with regulations 3, 4, and 5 being clamped in one simulation each. If all three simulations show disruption of rhythmicity loop II is regarded as essential. On the right: Clamping the combination of loop I and II. If all six simulations show disruption of rhythmicity the combination of loop I+II is regarded as essential. If both single loops I and II are essential, we denote this as I $\vee$ II, i.e. interrupting any loop stops oscillations. If the combination of loop I and II is essential, we denote it as I $\wedge$ II, i.e. interrupting both loops simultaneously stops oscillations.

generates oscillations and vice versa. In analogy to the single loop case we define a combination of loops as essential, if separate clamping of all pairwise combinations of regulations from the two loops leads to disruption of circadian rhythmicity. An example is given in Figure **S5-1**.

If two single loops were essential, then the combination of both loops is also essential. Therefore we do not need to test the combinations of loops that have already been identified as single essential loops. If a combination of loops

is essential, but not the single loops, these loops mutually compensate for loss of one of the loops.

An overview over the procedure is shown as pseudocode in Algorithm 1.

Algorithm 1 targeted clamping

```
loop_list ← list negative feedback loops                    ▷ loop is a set of edges
essential_loops ← empty_list()
for all k ∈ 1 ... max_level do            ▷ combinations of up to max_level loops
    for all comb ∈ combinations of k loops do
        if ¬any{el ⊆ comb | el ∈ essential_loops, el ≠ ∅} then
            rhythmic ← FALSE
            for all clamp_edges ∈ cartesian_product(comb) do
                time_series ← simulate_model(clamp_edges)
                rhythmic ← rhythmic ∨ is_rhythmic(time_series)
            if rhythmic then
                essential_loops.append(comb)
return essential_loops
```

Output notation and oscillators

The algorithm returns a list of feedback loops needed to be clamped to disrupt rhythmicity. We denote this in the form of a logic function. For the example in Figure **S5-1**A, we could have for instance $I \vee II$ (see Figure caption).

Assuming a more complex network with 5 negative feedback loops, we could also have:

$$system_{arrhythmic} = I \vee (II \wedge III) \vee (IV \wedge V)$$

with $I \dots V$ being 1 if the corresponding loop is clamped and 0 otherwise. It means that clamping loop I disrupts rhythms and also clamping the combination of loops II and III as well as loops IV and V, while clamping loop II or V alone does not.

This condensed description in Disjunctive Normal Form (DNF) is useful to see the different options of how to disrupt rhythms with a minimal amount of clamped regulations.

If we want to see on the other hand, which minimal amount of loops is necessary to generate rhythms, we can transform the function (by negating, applying De Morgans law and converting back to DNF):

$$
\begin{aligned}
system_{rhythmic} = &(\neg I \wedge \neg II \wedge \neg IV) \vee (\neg I \wedge \neg II \wedge \neg V) \\
&\vee (\neg I \wedge \neg III \wedge \neg IV) \vee (\neg I \wedge \neg III \wedge \neg V)
\end{aligned}
$$

$\neg I \ldots \neg V$ are 1 if the corresponding loop is active and 0 otherwise. The expression now describes, which feedback loops need to be active in order for the system to be rhythmic.

In other words: each combination of loops inside the brackets (e.g. I, II and IV) constitutes a minimal oscillator. Thus, all brackets contain minimal sets of loops that can generate oscillations in synergy.

We identify minimal oscillators for all logic functions and verify them in separate simulations. Thus, we are able to find necessary and sufficient conditions for rhythms in an automated way in all fitted models.

Loop frequencies in different tissues

We used our clamping analysis to find essential loops in all fitted models. Interestingly a large variety of loops and oscillators was found (see main text). Figure **S5-2** shows the relative frequencies of loops found in 10 tissues. To keep the legend simple, essential loops were counted if they were present in the logic function determined by our method (see above), regardless of their position in this function.

References

[1] Pett JP, Korenčič A, Wesener F, Kramer A, Herzel H. Feedback loops of the mammalian circadian clock constitute repressilator. PLoS Computational Biology. 2016;12(12):e1005266.

[2] Thomas R, Thieffry D, Kaufman M. Dynamical behaviour of biological regulatory networks–I. Biological role of feedback loops and practical use of the concept of the loop-characteristic state. Bull Math Biol. 1995;57:247–276.

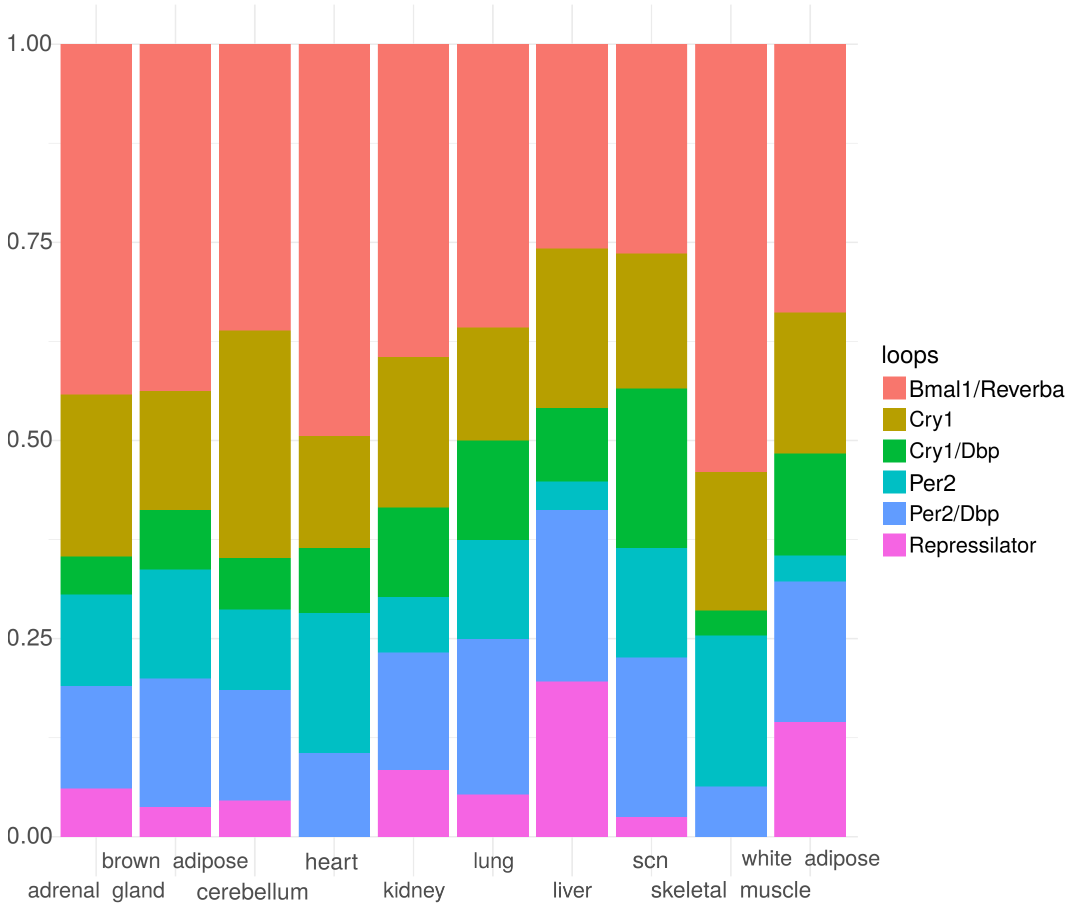

Figure S5-2: Relative loop frequencies in 10 tissues. In skeletal muscle and heart no repressilator was found, but they have a large fraction of models with essential *Bmal1*-*Rev-erb-*α loop. In SCN the fraction of combinations with *Per2* and *Cry1* loops is particularly large and most repressilators were found in liver.
