# Supplementary material for: Co-existing feedback loops generate tissue-specific circadian rhythms
Source: Life Sci Alliance. 2018 Jun 14;1(3):e201800078. doi: 10.26508/lsa.201800078 (PMC6238625; doi:10.26508/lsa.201800078)
Supplement: Supplementary file 5 [file LSA-2018-00078_Supplementary_Information_4.pdf]

S4 Finding initial estimates with Vector Field Optimization (VFO)

Fitting the vector field

In our case we have five differential equations, with associated experimental time series. We want to optimize the parameters of these differential equations, such that the solution resembles the time courses. Usually for complex sets of equations this is done with numerical simulations for each parameter set.

With Vector Field Optimization (VFO), however, we aim at fitting the vector field in regions where experimental data is available. Thus, we make use of the situation that time courses exist for all equations.

Fitting is done by plugging in time series values in the right hand side of the differential equations and time series derivative values at the left hand side. This is schematized in Example **S4-1**. Instead of the raw experimental time series, we use harmonic fits (as described in Supplement S3) as well as their analytic derivatives.

Example S4-1 Plugging in time course values for VFO. In this toy differential equation with an inhibition-, activation- and degradation term the time series values for fits of genes A, B and C are plugged in at the blue places. The time derivative of the time series fit for gene A is plugged in at the green place. Then, for each set of parameter values (red) the difference between the left and right side of the equation is used as a score (sum of squared errors) and the score is minimized using a bounded gradient method. In this way estimates of the unknown parameters (red) are obtained.

$$\frac{d[A]}{dt} = \underbrace{\left(\frac{1}{\frac{[B]_\tau}{\mathrm{inh}} + 1}\right)^m}_{\text{inhibition}} \cdot \underbrace{\left(\frac{\frac{max\_act \cdot [C]_\tau}{\mathrm{act}} + 1}{\frac{[C]_\tau}{\mathrm{act}} + 1}\right)^n}_{\text{activation}} \underbrace{-\mathrm{d} \cdot [A]}_{\text{degradation}}$$

Then, parameters are chosen to minimize the squared differences between both sides of the equation. Since no numeric simulation is required, evaluation

of one parameter set is fast. To keep our restrictions on parameter ranges, a bounded gradient method is used for optimizing parameter values.

Finding initial estimates

We use VFO only for optimizing initial model parameter sets. In principal it is possible to fit a model solely with VFO (if the differential equations can exactly resemble the time courses used for fitting). However, there are shortcomings of this method:

If the fit is not perfect and right hand side and the derivative on the left hand side deviate for one or more equations, it is not guaranteed that the vector field forms asymptotically the desired limit cycle at the respective position. Tests with numeric simulations showed that in some of the optima it indeed a limit cycle is formed, in others, however, the gene levels go to a steady state.

We could increase the frequency of found limit cycles by introducing a "transient trick", requiring vectors in the vicinity of the desired cycle in the vector field to point towards the cycle. However, there were always cases of failure and the resemblance of found limit cycles with time series was overall not sufficient.

Nevertheless, VFO directs parameters to regions in parameter space where time courses and equations roughly match. We therefore tested the use of VFO to compute starting conditions for optimization with a second method. The solutions found with VFO are also used to center initial parameter ranges for the second method around the VFO estimates.

In our implementation we use Particle Swarm Optimization (PSO) as a second method. We find that VFO+PSO yields significantly better scores for liver and SCN than PSO alone (main text Figure 4B), while the scores are comparable in kidney and adrenal gland. Notably, almost no good fits were found in SCN without using VFO. There were no marked differences in the loop distribution when using PSO alone or in combination with VFO.

Further, we use different scoring functions for VFO and PSO that possibly complement each other. While the scoring function used for PSO only optimizes period, phase relations and fold changes, VFO also accounts for the curve shape. VFO finds regions in parameter space, taking the differences between time courses carefully into account, but possibly failing to fit a real limit cycle. Then, PSO is used including numeric simulations to guarantee oscillations with a period of 24 h, correct phase relationships and fold changes of gene expression levels.

Parameter distribution after VFO

VFO determines starting conditions for PSO and directs the search to certain regions in parameter space. We use the computed result of VFO to initialize 1 of 70 particles used in PSO, while the others are still initialized randomly using latin hypercube sampling. (The score of the VFO particle is mostly very good and pulls other particles into its direction.)

To assess the effect of VFO, we ran 50 optimizations with only VFO on the liver data set and examined the resulting starting estimates (see Fig. **S4-1**). 37 of 50 of these starting estimates were already oscillating and 7 had scores below 10 using the PSO scoring function. Interestingly, the distributions of many parameters are quite restricted within the allowed ranges, suggesting specific optimal values.

For example the delays of *Bmal1* (del1) and *Rev-erb-*α (del2) are about 6 and 1 respectively. This relation is consistent with the time gap between peaks of those genes and with the *Bmal1*-*Rev-erb-*α feedback loop in particular. Further the kinetic parameters corresponding to this loop (ar1, b_RevErb and ba2) are also contained in narrow ranges. Indeed, our clamping analysis revealed that *Bmal1*-*Rev-erb-*α loops are prevalent.

The emergence of such specific parameter patterns suggests that it is reasonable to pre-emphasize the search for optima with VFO. Improved scores and pre-selection of parameter sets indicate, that VFO is well suited to improve optimization.

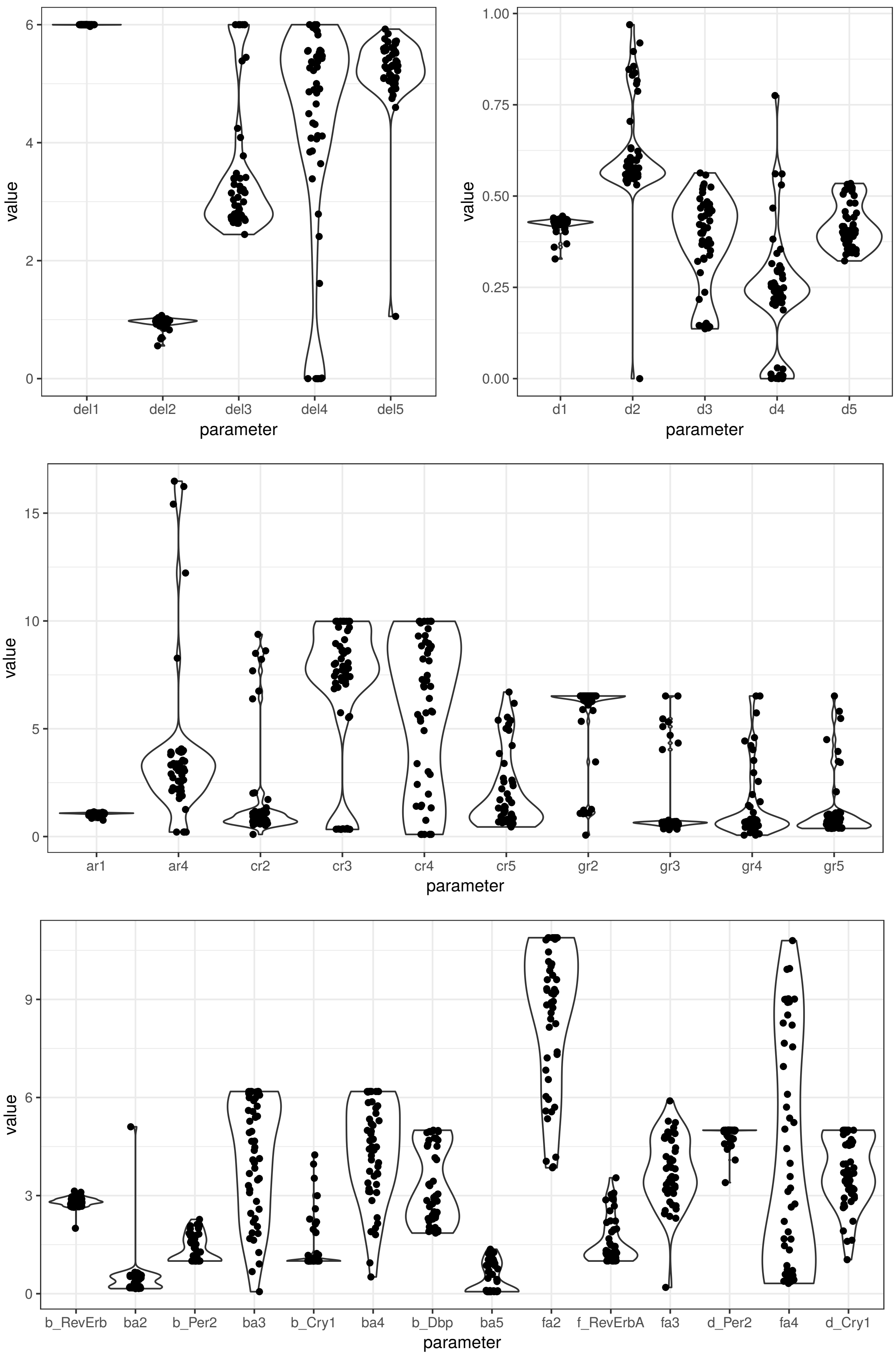

Figure S4-1: Parameters after VFO. The resulting parameter distributions deviate clearly from equidistributions in the alowed parameter ranges such as $[0, 6]$ for delays and $[0, 1]$ for degradation rates.
