# Supplementary material for: Co-existing feedback loops generate tissue-specific circadian rhythms
Source: Life Sci Alliance. 2018 Jun 14;1(3):e201800078. doi: 10.26508/lsa.201800078 (PMC6238625; doi:10.26508/lsa.201800078)
Supplement: Supplementary file 6 [file LSA-2018-00078_Supplementary_Information_6.pdf]

S6 Complex Dynamics via Combinatorial Regulations

Harmonics and tori in good parameter fits

Due to noise and a small number of replicates, the curve shape of experimentally measured mRNA expression time courses is quite variable. Using VFO (Supplement S4) to pre-emphasize parameter values before optimization involves also the curve shapes of harmonic fits. However, the scoring function used for final optimization only includes period, phases and fold changes (see Supplement S3).

Our gene regulatory model involves combinatorial regulation and multiple nonlinearities. Thus, complex waveforms and attractors are possible. It has been discussed earlier [1, 2] that combinatorial transcriptional regulation can generate multimodal waveforms with pronounced harmonics. Furthermore, multiple negative feedback loops might lead to superpositions of independent oscillations termed tori.

Thus, complex curve shapes, harmonics and tori are in principal not excluded as long as phases and amplitudes fit well. Nevertheless, the vast majority of fitted models generates time courses with only a single peak in their power spectrum and unimodal waveforms resembling the data.

Examples

Harmonics were also described for the 5-gene DDE model in the original publication [3] and a torus was observed in [4].

An example of a fitted model with harmonics is shown in Figure **S6-1**. Harmonics were more often observed for genes and tissues with particularly small fold changes in the data, such as *Cry1* and *Per2* in the SCN.

A torus is shown in Figure **S6-2**.

References

[1] Westermark PO, Herzel H. Mechanism for 12 hr rhythm generation by the circadian clock. Cell reports. 2013;3(4):1228–1238.

[2] Korenčič A, Bordyugov G, Košir R, Rozman D, Goličnik M, Herzel H. The interplay of cis-regulatory elements rules circadian rhythms in mouse liver. PLoS One. 2012;7:e46835.

[3] Korenčič A, Košir R, Bordyugov G, Lehmann R, Rozman D, Herzel H. Timing of circadian genes in mammalian tissues. Sci Rep. 2014;4:5782.

[4] Pett JP, Korenčič A, Wesener F, Kramer A, Herzel H. Feedback loops of the mammalian circadian clock constitute repressilator. PLoS Computational Biology. 2016;12(12):e1005266.

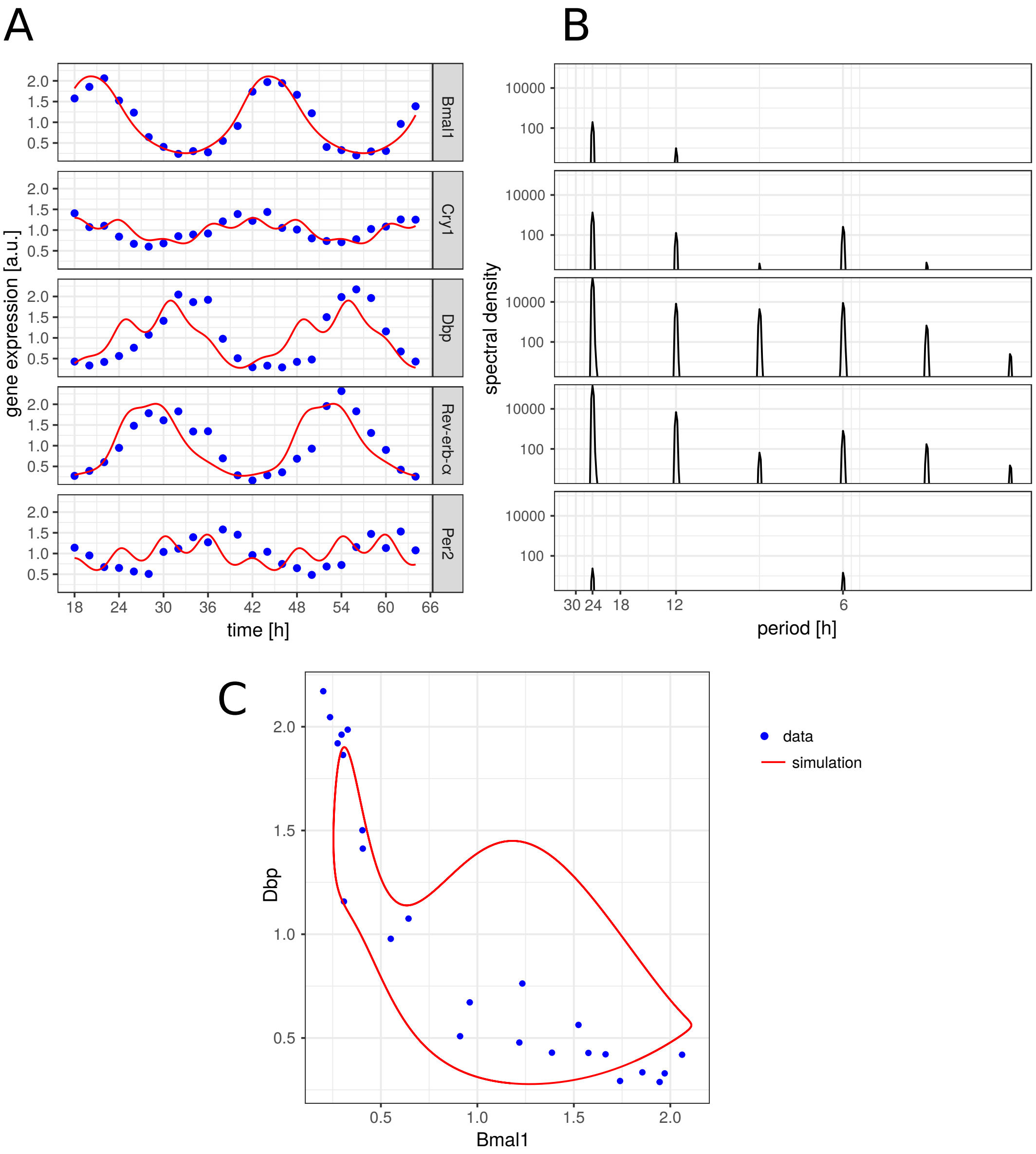

Figure S6-1: Simulation of a model resembling expression data from adrenal gland. (A) Simulated time series (red) and data points (blue). (B) Power spectrum of the simulated time series. Note the harmonics at 12, 8 and 6 h. (C) Phase-portrait showing a limit cycle.

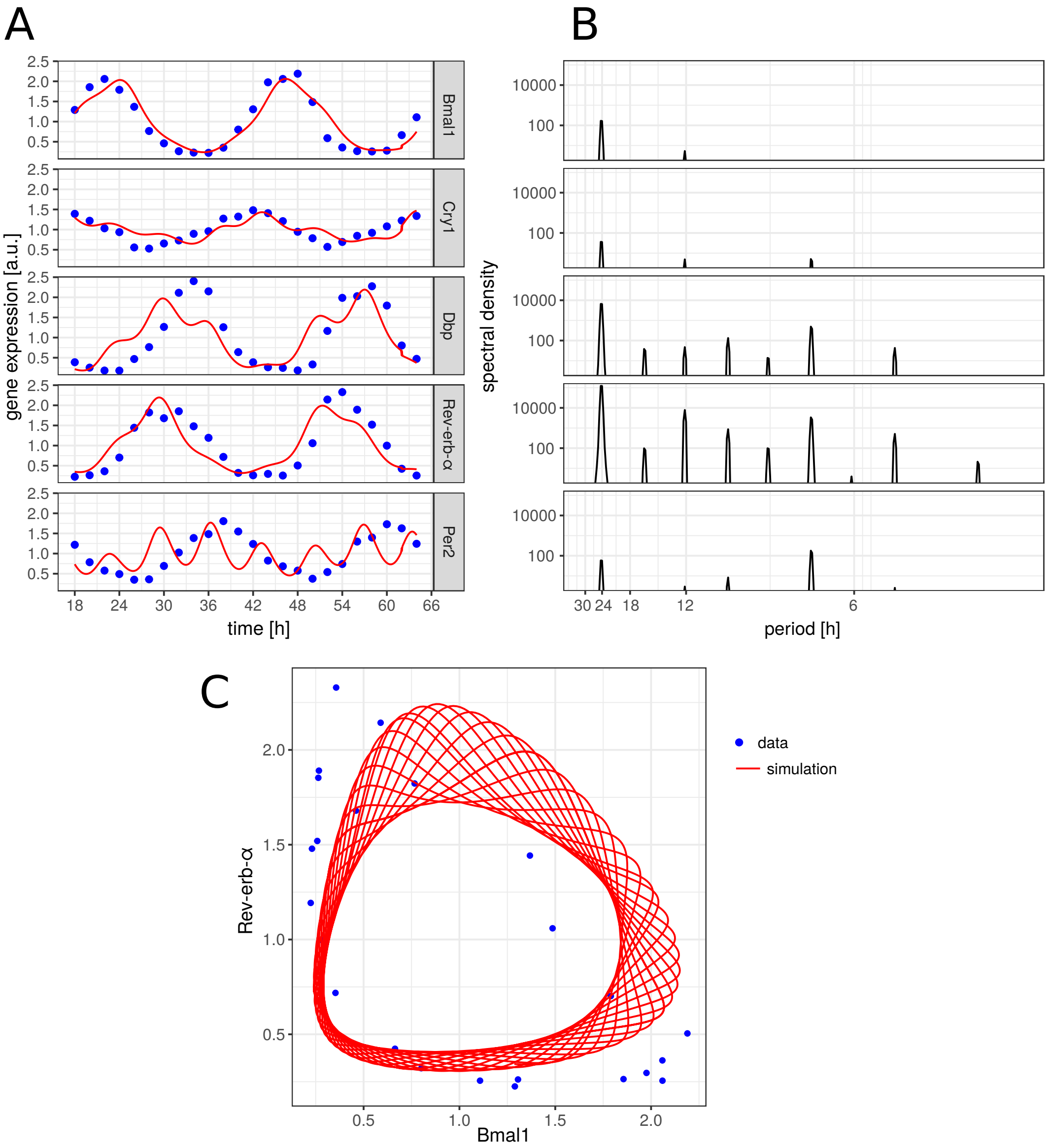

Figure S6-2: Simulation of a model resembling expression data from kidney. (A) Simulated time series (red) and data points (blue). (B) Power spectrum of the simulated time series. Note the peaks at about 7 and 10 h. (C) Phase-portrait showing a torus.
